# Photo‐Clickable Triazine‐Trione Thermosets as Promising 3D Scaffolds for Tissue Engineering Applications

**DOI:** 10.1002/adhm.202401202

**Published:** 2024-07-17

**Authors:** Åshild Johansen, Jinjian Lin, Shuntaro Yamada, Samih Mohamed‐Ahmed, Mohammed A. Yassin, Cecilie Gjerde, Daniel J. Hutchinson, Kamal Mustafa, Michael Malkoch

**Affiliations:** ^1^ Center of Translational Oral Research (TOR) Tissue Engineering Group Department of Clinical Dentistry University of Bergen Årstadveien 19 Bergen 5009 Norway; ^2^ School of Engineering Sciences in Chemistry Biotechnology and Health (CBH) Department of Fibre and Polymer Technology Division of Coating Technology KTH Royal Institute of Technology Teknikringen 56 Stockholm SE‐100 44 Sweden

**Keywords:** biocompatibility, regenerative medicine, thermoset, thiol‐ene, thiol‐yne, tissue engineering, triester‐triazine‐trione

## Abstract

There is an overwhelming demand for new scaffolding materials for tissue engineering (TE) purposes. Polymeric scaffolds have been explored as TE materials; however, their high glass transition state (T_g_) limits their applicability. In this study, a novel materials platform for fabricating TE scaffolds is proposed based on solvent‐free two‐component heterocyclic triazine‐trione (TATO) formulations, which cure at room temperature via thiol‐ene/yne photochemistry. Three ester‐containing thermosets, TATO‐1, TATO‐2, and TATO‐3, are used for the fabrication of TE scaffolds including rigid discs, elastic films, microporous sponges, and 3D printed objects. After 14 days’ incubation the materials covered a wide range of properties, from the soft TATO‐2 having a compression modulus of 19.3 MPa and a T_g_ of 30.4 °C to the hard TATO‐3 having a compression modulus of 411 MPa and a T_g_ of 62.5 °C. All materials exhibit micro‐ and nano‐surface morphologies suited for bone tissue engineering, and in vitro studies found them all to be cytocompatible, supporting fast cell proliferation while minimizing cell apoptosis and necrosis. Moreover, bone marrow‐derived mesenchymal stem cells on the surface of the materials are successfully differentiated into osteoblasts, adipocytes, and neuronal cells, underlining the broad potential for the biofabrication of TATO materials for TE clinical applications.

## Graphical abstract

In this study, researchers propose a solvent‐free approach for the fabrication of novel scaffolding materials using two‐component heterocyclic triazine‐trione formulations, which cure in seconds at room temperature via thiol‐ene/yne photochemistry. The materials are suitable for bone tissue engineering, featuring excellent cytocompatibility, fast cell proliferation, and differentiation of bone marrow mesenchymal stem cells into osteoblasts, adipocytes, and neuronal cells, while minimizing cell apoptosis and necrosis.

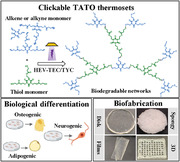

## 1 Introduction

The demand for organs and tissues for transplantation has surged, driven by the imperative to rectify or substitute defects in biological tissue arising from conditions such as infection, oncogenic diseases, trauma, or congenital anomalies.^[^ ^1^, ^2^ ^]^ Addressing the need for biological tissue, the field of tissue engineering arose in the early 1990s, dedicated to the creation of synthetic tissue implants. Driven by medical, academic, and commercial interests, the ultimate goal is to engineer functional tissues for implantation in the body, with the ability to perform the biological tasks needed at the recipient sites.^[^ ^1^, ^3^ ^]^

The success of tissue engineering strategies hinges on several crucial elements within its paradigm, with a central emphasis on scaffolding biomaterials, often in conjunction with cells and/or signaling molecules.^[^ ^1^, ^4^ ^]^ Scaffolds serve as temporary biomaterial frameworks, mimicking the extracellular matrix (ECM), within which cells can be seeded. The material used serves as the foundation for scaffolds and determines their mechanical, chemical, and biological properties. Several key considerations include, but are not limited to, biocompatibility, optimal mechanical properties, biodegradability, sterility, and formability.^[^ ^5^ ^]^ Regardless of tissue types to be engineered, the materials need to support cell adhesion, proliferation and differentiation over their surfaces. Scaffolding biomaterials used in tissue engineering are mostly categorized into metals, ceramics, and polymers, of either synthetic or natural origin, or composite materials.^[^ ^4^ ^]^ Synthetic biodegradable polymers have gained a prominent role in the biomedical field in different tissue engineering applications due to their versatility and superior mechanical properties compared to their natural‐origin counterparts.^[^ ^6^ ^]^ Despite notable advancements in recent years, the current landscape of biomaterials falls short of meeting the diverse and evolving demands of tissue engineering and regenerative medicine. For example, while polyester‐ and polycarbonate‐based biomaterials have been widely applied in tissue engineering, certain limitations remain, which include a lack of flexibility in formability at ambient temperature without the use of solvent, sufficient mechanical strength and elasticity, and biocompatibility.^[^ ^7^ ^]^ Therefore, there is an imperative need for the development of an entirely new category of biomaterials that serve as innovative tissue engineering scaffolds.

In recent years, the family of 1,3,5‐triazine‐triones (TATO) has garnered significant attention as promising candidates in the field of biomedical applications. This class of compounds has demonstrated notable potential across a spectrum of biomedical domains, including anti‐protozoal,^[^ ^8^, ^9^, ^10^ ^]^ anti‐cancer,^[^ ^11^, ^12^, ^13^, ^14^, ^15^, ^16^, ^17^, ^18^ ^]^ anti‐malarial,^[^ ^19^, ^20^, ^21^, ^22^ ^]^ anti‐viral^[^ ^23^ ^]^ purposes. Many of these have already been approved by the U.S. Food and Drug Administration (FDA) for medical use, underscoring their translational relevance.^[^ ^24^ ^]^

Expanding the versatility in biomedical contexts, researchers have successfully engineered and applied strong and biocompatible TATO polymeric networks to act as bone fracture fixation materials or dental restoration composites. This has been accomplished by stoichiometrically mixing thiol and alkene or alkyne trifunctional TATO monomers together with hydroxyapatite (HA) fillers and then crosslinking the monomers into 3D networks through high‐energy visible light induced thiol‐ene (HEV‐TEC) or thiol‐yne (HEV‐TYC) “click” chemistry.^[^ ^25^, ^26^, ^27^, ^28^ ^]^ The TEC and TYC reactions have many viable advantages suited to biomedical applications, including high chemoselectivity between the monomers, the ability to proceed in surgical environments and in close vicinity to tissue, and high monomer conversion, which effectively reduces the leaching out of monomers.^[^ ^29^, ^30^, ^31^, ^32^, ^33^ ^]^ This is in stark contrast to biomaterials based on acrylate monomers, where ill‐defined thermosets such as poly‐methyl methacrylate (PMMA) or gelatin methacrylate (GelMA) result in monomer leach out and damage to tissues surrounding the device. Moreover, by carefully designing the TATO monomers, with respect to the type of chemical bonds and their distance to the heterocyclic ring, the crosslinking density of the biomaterials can alter the mechanical properties of the material. Notably, TATO monomers are commercially available or can be produced in large quantities making scalability possible, which is beneficial for their future clinical applications.

Recently, we have successfully synthesized TATO monomers that combine ester or amide linkages with alkene or alkyne functionalities. These monomers were designed to introduce biodegradability and flexibility to HA‐infused TATO composite materials, in order to broaden their scope in biomedical applications.^[^ ^34^ ^]^ The introduction of ester linkages to the TATO‐alkene and alkyne structures resulted in significant softening of the crosslinked composite materials; substantially reducing the brittleness and modulus when compared to a previously established composite constructed from 1,3,5‐triallyl‐1,3,5‐triazine‐2,4,6‐trione (TATATO), tris[2‐(3‐mercaptopropionyloxy)ethyl]isocyanurate (TEMPIC) and HA. The TATO resins are viscous mixtures that harden effectively at room or body temperature with HEV‐TEC or TYC chemistry. This allows for the resins to be applied, shaped and cured in situ without damage to surrounding living tissues. This is a considerable benefit, compared to other synthetic polymers with high glass transition temperatures (T_g_), such as polycaprolactone (PCL), polylactic acid (PLA) and poly(lactic‐co‐glycolic) acid (PLGA), which require high temperatures or the addition of solvents to be shaped, which limits their application as TE materials.^[^ ^35^, ^36^ ^]^

**Figure 1:**
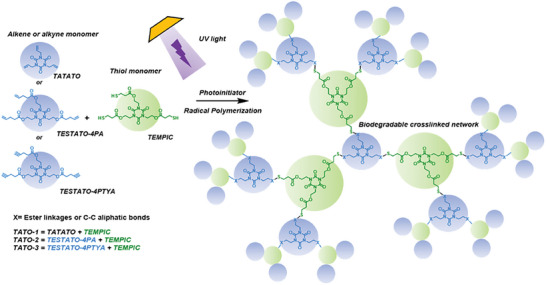
Schematic of the formulation of the TATO‐1, TATO‐2, and TATO‐3 materials. Undergoing fast light‐induced TEC or TYC radical polymerization, a crosslinked network is fabricated within seconds.

In this study, we explore three ester‐enriched thermosets, namely TATO‐1, TATO‐2, TATO‐3, suited as potential biomaterials for tissue engineering applications (**Figure** **1**). These thermosets are constructed from TEMPIC together with either TATATO, or the ester containing alkene or alkyne TATO monomers TESTATO‐4PA or TESTATO‐4PTYA. A systematic examination of the thermosets has been conducted, encompassing mechanical characteristics, surface morphology, chemical attributes, and degradability. This comprehensive analysis contributes to a profound understanding of the intrinsic nature of the ester‐incorporated crosslinked thermoset networks. Moreover, the study extends to the evaluation of these materials' biocompatibility, their functionality as supportive matrices for growth and multi‐lineage differentiation of mesenchymal stem cells (MSC), and their adaptability for scaffold fabrication. The findings of this study introduce, for the first time, a novel class of ester‐abundant thermoset materials, showcasing their potential as promising candidates for tissue engineering across a spectrum of regenerative applications.

## 2 Results and Discussion

### 2.1 Surface Properties at Micron and Nano Scale of TATO Materials

**Figure 2:**
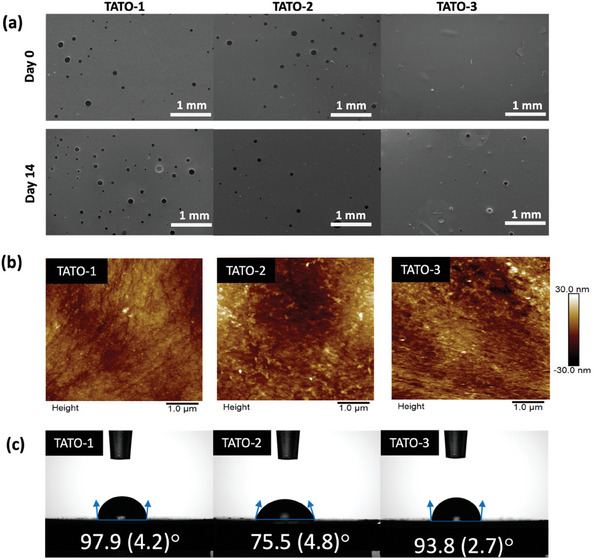
Surface characterizations of the TATO materials. a) SEM images at day 0 and day 14 after PBS (pH = 7.4) treatment; b) AFM images of the baseline materials; c) Contact angles of the baseline materials. *n* ≥ 5 for each material, results presented as mean (SD).

The TATO‐1, TATO‐2 and TATO‐3 materials were prepared via photo‐initiated TEC or TYC radical polymerization, resulting in crosslinked thermoset networks (Figure 1). The choice of C‐C unsaturated monomers led to differences in the concentration of ester groups within the material and the crosslinking density, with the TATO‐1, TATO‐2 and TATO‐3 materials having theoretical ester concentrations of 3.849, 5.783, and 5.683 mmol g^−1^, respectively. Previous DMA results showed that the crosslink density of the three materials was 12.3 (0.9), 10.0 (0.6), and 15 (0.2) M_c_, respectively.^[^ ^34^ ^]^ Rheological measurements were made of each material to determine the viscosity of each resin at room temperature. The viscosity versus shear rate data showed that the viscosity increased in the order TATO‐2 < TATO‐1 < TATO‐3 (Figure S2a, Supporting Information). Scanning electoron microscopy (SEM) characterization of the three materials’ surfaces revealed that on a micron scale they all showed similar flat patterns with the presence of air bubbles (**Figure** **2**a). These bubbles occurred due to air being trapped in the resins during the mixing of the formulations. Histogram results analyzed by ImageJ demonstrated that there were more bubbles present on the surface of TATO‐1 than TATO‐2, which matched with TATO‐1 having the higher viscosity (Figure S2b, Supporting Information).^[^ ^37^ ^]^ TATO‐3, however, had the lowest concentration of air bubbles on its surface, despite having a significantly higher viscosity than the other two materials. This was likely due to increased liberation of air from TATO‐3 during the early stages of curing, due to both the longer HEV exposure required to cure TATO‐3 and the more exothermic nature of the TYC reaction compared to the TEC reactions of TATO‐1 and TATO‐2.^[^ ^38^ ^]^ Atomic force microscopy (AFM) images revealed nano‐scale morphological similarities across all thermoset surfaces (**Figure** **2**b). The roughness analysis (Ra) showed values of 4.52 (1.08), 6.52 (0.37), and 5.72 (2.92) nm for TATO‐1, TATO‐2, and TATO‐3, respectively. TATO‐2 exhibited the highest roughness with the smallest standard error, indicating consistent curing. TATO‐3, with a slightly higher standard error, showed varied surface features, possibly due to demanding curing conditions at which a higher amount of photo‐initiator and curing time were required for the full conversion of the TESTATO‐4PTYA and TEMPIC monomers.^[^ ^34^ ^]^ The harsher curing conditions likely led to a non‐synchronic radical polymerization at different locations of the uncured TATO‐3 resin and thus, higher surface variance of the discs prepared with TATO‐3.

Contact angle measurements indicated that both TATO‐1 and TATO‐3 were hydrophobic, with average contact angles of 97.9 (4.2) and 93.8 (2.7)°, respectively (Figure 2c). In contrast, TATO‐2 exhibited hydrophilic behavior, with a contact angle of 75.5 (4.8)°. The variations in hydrophilicity may have arisen from differences in chemical structures, particularly the density of ester linkages and crosslinking density of the three materials. The higher ester density in TATO‐2 facilitated stronger hydrogen bonding with water, resulting in significantly higher hydrophilicity compared to TATO‐1, which had a similar crosslinking density. Although TATO‐2 and TATO‐3 had similar theoretical ester densities, their hydrophilicity deviated, likely due to differing crosslinking densities. Higher crosslinking density in TATO‐3 impeded esters from forming hydrogen bonds with water, making it more hydrophobic. In contrast, TATO‐2, with two‐thirds of TATO‐3′s crosslinking density, provided easier access for esters to water, creating a more hydrophilic environment.

### 2.2 Mechanical Properties of the TATO Materials

**Figure 3:**
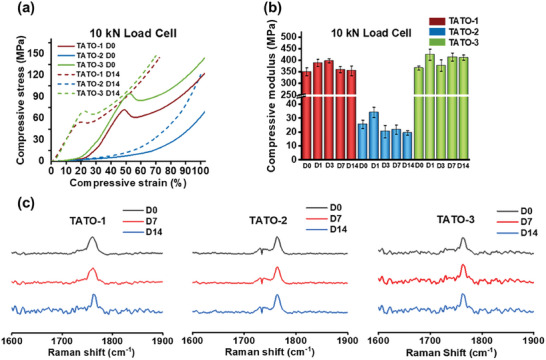
Mechanical results for the prepared discs under different time points as well as Raman spectroscopy results. a) Compressive behavior under 10 kN load cell; b) Compressive modulus versus maximum compressive strain under 10 kN load cell.; c) Raman spectroscopy results for the TATO materials under different time points with PBS treatment (0, 7, and 14 days), focusing on the peak at 1780 cm^−1^ corresponding to the carbonyl signal of the TATO ring and the ester groups. For (b), *n* = 5 for each material and time point, data presented as mean with error bars representing SD.

The mechanical properties of the TATO‐1, TATO‐2 and TATO‐3 materials before (day 0) and after incubation in PBS (pH = 7.4) at 37 °C (day 1, 3, 7, and 14) were characterized using compression testing with a 10 kN load cell in order to reach a large deformation (**Figure** **3**, **Table** **1**). While the TATO‐1 and TATO‐3 materials reached a failure point, the TATO‐2 material modulus kept increasing until 100% compressive strain was reached. Such a high strain, which corresponded to a load of 10 kN (1019.72 kg) would not be realistic in vivo; therefore, the modulus of TATO‐2 was taken as the modulus at the yielding strain of the TATO‐3 material for the sake of comparison between the three materials.^[^ ^39^ ^]^ Under dry conditions, the compressive stress versus strain results (Figure 3a,b and Table 1) demonstrated that materials TATO‐1 and TATO‐3 yielded at compressive strains of 50.2 (2.2) and 50.5 (1.7) %, respectively. Meanwhile, TATO‐1 and TATO‐3 showcased compressive modulus of 349.9 (17.7) and 366.2 (8.9) MPa at failure, respectively. Both these values were slightly higher than the modulus of bulk PCL, which is between 299 and 317 MPa under dry conditions (39). For TATO‐2, its modulus at the yield point of TATO‐3 was calculated to be 25.4 (3.0) MPa, which is 13.8, 14.4 and 12.1 times smaller than TATO‐1, TATO‐3 and bulk PCL, respectively. After 14‐days incubation in PBS (pH = 7.4) buffer, the strain at failure of TATO‐1 and TATO‐3 drastically reduced 2.4 and 2.2 times to 21.2 (2.1) and 22.6 (1.1)%, respectively, while their modulus remained mostly unchanged.

**Table 1:** Mechanical properties and thermal analysis of the TATO thermosets under dry or wet conditions (immersion in PBS at 37 °C for 0, 1, 3, 7, or 14 days). All values given as mean with standard deviation (SD) in parentheses (*n* ≥ 5).

Materials	Time immersed in PBS at 37 °C (days)	E_C_ [MPa] [10 kN]	ε_c_ at failure [%] [10 kN]	T_g_ [°C]	Onset Point [°C]	Ester density [mmol g^−1^, theoretical]	Contact angle [°]	Height [nm] (AFM)	R_a_ [nm] (AFM)	Crosslink density M_c_*	Water absorption [%] *
TATO‐1	0	349.9 (17.7)	50.2 (2.2)	71.1 (0.6)	61.3 (0.8)	3.849	97.9 (4.2)	21.7 (3.9)	4.5 (1.1)	12.3 (0.9)	1.30 (0.04)
	1	388.2 (15.5)	25.6 (2.1)	59.5 (0.2)	37.4 (1.9)						
	3	397.2 (9.4)	24.8 (1.2)	56.5 (1.7)	38.9 (2.9)						
	7	359.4 (12.7)	20.9 (1.3)	58.6 (3.1)	42.0 (2.8)						
	14	354.6 (21.2)	21.1 (2.1)	61.5 (0.3)	40.6 (2.1)						
TATO‐2	0	25.4 (3.0)	‐ (‐)	34.7 (0.4)	24.5 (0.4)	5.783	75.5 (4.8)	34.4 (2.2)	6.5 (0.4)	10.0 (0.6)	1.55 (0.03)
	1	34.0 (3.6)	‐ (‐)	22.5 (0.2)	7.4 (0.6)						
	3	20.3 (4.4)	‐ (‐)	23.5 (0.7)	6.9 (0.7)						
	7	21.6 (3.5)	‐ (‐)	25.2 (0.7)	6.7 (0.8)						
	14	19.3 (1.6)	‐ (‐)	30.4 (0.4)	10.3 (0.8)						
TATO‐3	0	366.2 (8.9)	50.5 (1.7)	60.9 (0.4)	48.5 (1.0)	5.683	93.8 (2.7)	35.9 (21.3)	5.7 (2.9)	15.0 (0.2)	1.37 (0.05)
	1	423.7 (24.3)	27.8 (1.7)	63.9 (0.9)	38.1 (2.0)						
	3	388.5 (17.3)	25.2 (3.8)	63.4 (3.4)	43.4 (3.7)						
	7	414.0 (17.5)	25.3 (2.2)	61.7 (4.2)	40.5 (5.1)						
	14	411.4 (10.9)	22.6 (1.1)	62.5 (1.2)	46.4 (3.9)						

^*^results reported in previous work.^[^ ^34^ ^]^

DMA results showed that the immersion in PBS buffer solution had the most significant impact on the mechanical properties of TATO‐1, resulting in its T_g_ decreasing from 71.1 (0.6) on day 0 to 61.5 (0.3) °C on day 14, and its onset point decreasing 20 °C from 61.3 (0.8) to 40.6 (2.1) °C (Table 1). Meanwhile, the T_g_ and onset point of the TATO‐3 materials were largely unaffected by immersion in PBS, showing values of 62.5 (1.2) and 46.4 (3.9) °C on day 14, respectively. The T_g_ and onset point of TATO‐3 were similar to that of TATO‐1 after 14 days in PBS; however, the TATO‐2 material had a much lower T_g_ and onset point of 30.4 (0.4) and 10.3 (0.8) °C, respectively, after 14 days in PBS incubation, which explained its rubber‐like compressive behavior below physiological temperature. All thermosets were monitored by Raman spectroscopy during their 14‐day immersion in PBS (Figure 3c). None of the spectra showed changes from hydrolysis of the ester linkages. The only carbonyl signal at all time points was at 1780 cm^−1^, which corresponded to the carbonyl groups from the TATO ring and the ester linkages. There were no emergent signals due to carboxylic acid groups, which would have been expected at ≈1715 cm^−1^.

### 2.3 Biodegradability of the TATO Materials

**Figure 4:**
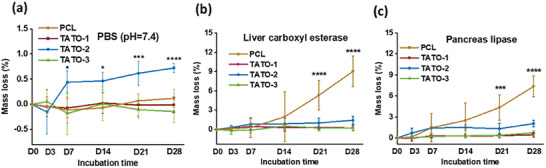
Hydrolytic and enzymatic degradation of PCL, TATO‐1, TATO‐2, and TATO‐3 materials in a) PBS, b) liver carboxyl esterase and c) pancreas lipase over 28 days. *n* = 5 for all materials and time points, data presented as mean with error bars representing SD, *p < 0.05, **p < 0.01, *** *p* < 0.001, **** *p* < 0.0001 (one‐way ANOVA).

Hydrolytic and enzymatic degradation were evaluated by incubating the thermosets in PBS with and without the presence of naturally derived esterase and lipase at physiological conditions. In PBS without enzyme, TATO‐2 showed significantly greater mass loss than TATO‐1, TATO‐3 and PCL after 28 days (**Figure** **4**a). TATO‐2 had lost 0.44 (0.25) % of its mass by day 7, increasing to 0.72 (0.11) % by day 28 (*p* = 0.0004 versus PCL; *p* < 0.0001 versus TATO‐1 and TATO‐3), while the other materials showed negligible mass loss in PBS. In the presence of esterase, while PCL lost ≈7.4 (1.7)% of its mass by 28 days in the presence of pancreas lipase (*p* < 0.0001 versus TATO‐1, 2, 3) and 9.0 (2.7) % in the presence of liver carboxylase (*p* < 0.0001 versus TATO‐1, 2, 3), TATO‐2 showed ≈2.1 (0.54) and 1.5 (0.67)% mass loss by pancreas lipase and liver carboxylase, respectively (Figure 4b,c). TATO‐1 and TATO‐3 seemed resistant to enzymatic biodegradation at given concentrations. The difference among the TATO materials was not statistically significant. The mass loss from the TATO materials occurred due to hydrolysis of the ester linkages as Raman spectroscopy showed complete monomer conversion upon TEC curing.^[^ ^34^ ^]^ TATO‐2 showed the highest degradation of the TATO materials due to its hydrophilic properties, its lower crosslink density and higher water absorption. Therefore, the degrading medium would have more readily absorbed into TATO‐2 than TATO‐1 or TATO‐3, resulting in higher mass loss. However, the mass loss of TATO‐2 due to biodegradation still proceeded at a slower speed compared to PCL. Further studies are needed using in vivo implantation models, where biomaterials are subjected to a combination of hydrolytic, enzymatic, and phagocytic degradation, as well as metabolic processes.^[^ ^40^, ^41^ ^]^ The degradation process is influenced by various patient‐specific factors, as well as the design of biomaterial architectures and scaffold fabrication methods. Additionally, material‐specific properties such as the degree of crosslinking, surface topography, and density play crucial roles. Tailoring the desired degradation rate is essential for specific applications. In the context of bone regeneration, for example, it is imperative that the degradation speed aligns with the rate of bone formation.^[^ ^41^ ^]^

### 2.4 In Vitro Indirect Test for Unspecific Cytotoxicity

**Figure 5:**
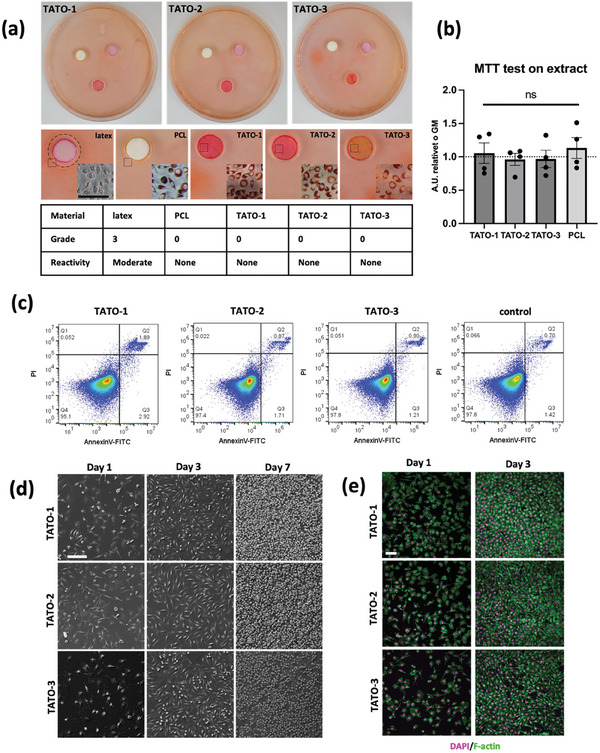
a) Macro‐ and microscopic images of cytocompatibility test following an agar diffusion test with TATO‐1, TATO‐2, TAT0‐3, PCL and latex. b) Measuring metabolic activity after 24‐h exposure to the extract of TATO‐1, TATO‐2, TATO‐3, and PCL. c) Flow cytometry of stained L929 cells with PI and AnnexinV after being attached to the TATO‐materials for 3 days. Plastic tissue culture plate served as control. d) SEM images of L929 cells seeded and cultured on TATO materials after 1, 3, and 7 days. e) Cytochemical staining with DAPI and Phalloidin of L929 cells seeded and cultured on TATO materials after 1 and 3 days. Scale bar = 100 µm. For (b), *n* = 5 for all materials, data presented as mean with error bars representing SD.

The in vitro unspecific cytotoxicity of the TATO materials was investigated through indirect contact test and extract analysis with reference to the ISO 10993–5:2009(E). The agar diffusion test showed no cytotoxic effect of the developed materials, comparable to the medical‐grade PCL with no detectable necrotic zone around or under the samples (Grade 0) (**Figure** **5**a, Table S1, Supporting Information). A qualitative assessment shows that the vital cells underneath the TATO‐discs had normal morphology as confirmed by the remaining Neutral Red stain incorporated in the cell's lysosomes. No difference in metabolic activity between the TATO materials and the negative control was detected (**Figure** 5b).

### 2.5 In Vitro Direct Contact Test for Unspecific Cytotoxicity

When L929 cells were cultured on the TATO materials, the viability of the cells was above 95% for all thermosets (Figure 5c). The sum of apoptotic and necrotic cells on TATO‐2 and TATO‐3 was ≈2%, comparable to the control. On TATO‐1, however, a slight increase in apoptotic and necrotic cells was found at 2.9 and 1.9%, respectively.

Cell adhesion is an essential step for adherant cells to survive and further to perform normal metabolism, proliferation and differentiation.^[^ ^42^, ^43^ ^]^ Despite the differences in material stiffness, the L929 cells managed to attach onto the TATO materials and showed typical fibroblastic morphology (Figure 5d,e). They proliferated and completely covered the material surface after 7 days. These findings confirm cytocompatibility of the TATO materials, strongly supporting the hypothesis that these materials are biocompatible and serve as suitable biomaterials for tissue engineering applications. The success of the novel materials as biomaterials in clinical practice relies, foremost, on their acceptance by the human body. It is therefore crucial to further investigate potential concerns, such as host responses and the impact of degradation byproducts through in vivo studies to ensure the comprehensive safety and efficacy of these materials.^[^ ^44^ ^]^

### 2.6 Viability and Growth of BMSC

**Figure 6:**
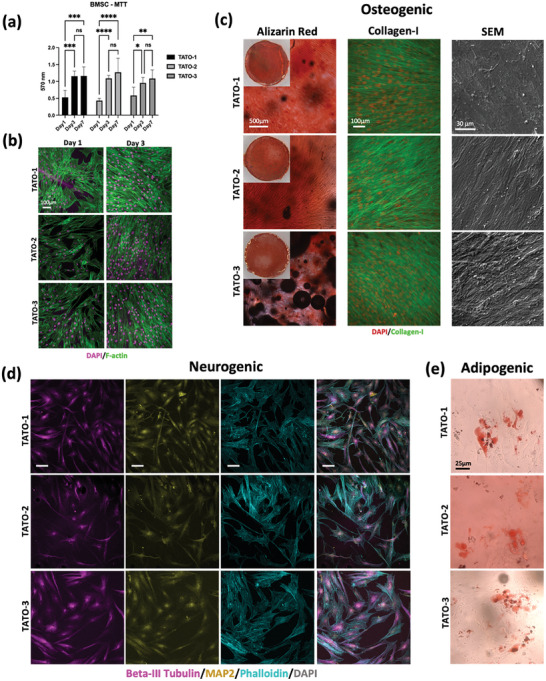
a) MTT of BMSC seeded on TATO materials and cultured in osteogenic medium up to 7 days b) Cytochemical staining with DAPI (nuclei = pink) and Phalloidin (F‐actin = green) of BMSC cells seeded and cultured with normal growth medium on TATO materials after 1 and 3 days. Scale bar = 100 µm c) Evaluating osteogenic differentiation with Alizarin red staining after 14 days, immunostaining of Collagen‐I (DAPI = nuclei in red) and SEM images with 2000× magnification. d) Immunostaining with neurogenic markers (Beta‐III Tubulin and MAP2) and Phalloidin. Scale bar = 100 µm. e) Oil Red O Assay staining fat droplets red. For (a), *n* = 5 for all materials and time points, data presented as mean with error bars representing SD, *p < 0.05, **p < 0.01, ***p < 0.001, ****p < 0.0001.

The attachment and growth of human bone marrow mesenchymal stem cells (BMSC) were also evaluated. The BMSC attached to the surface of the TATO materials with typical spindle‐like morphology and continued to grow until they completely reached confluency by day 7 (**Figure** **6**a,b). Cell attachment and growth are largely associated with surface hydrophilicity of the materials.^[^ ^43^ ^]^ Generally, cell attachment and metabolic activity are superior on hydrophilic surfaces with contact angles ranging from 40 to 90°, with minor differences between cell types.^[^ ^43^, ^45^, ^46^, ^47^, ^48^, ^49^ ^]^ Among the proposed materials, TATO‐2 was the only hydrophilic material by definition. Likewise, surface stiffness also influences BMSC attachment and growth.^[^ ^43^, ^50^, ^51^, ^52^ ^]^ Nonetheless, despite differences in wettability and stiffness among the TATO materials, no significant difference in cell attachment and growth was observed for the BMSC.

### 2.7 Multi‐Lineage Differentiation of BMSC on TATO Materials

The multi‐lineage differentiation of BMSC on the TATO materials was evaluated to explore potential applications in tissue engineering (Figure 6c–e and Figure S3, Supporting Information). In short, BMSC were able to differentiate into osteoblasts, neuronal cells, and adipocytes for all TATO materials, confirmed by the synthesis of collagen type 1 and biomineralization, expression of two neurogenic markers, MAP2 and Class III beta‐tubulin, as well as neuronal morphology, and the formation of lipid droplets, respectively. The stiffness and rigidity of the biomaterial guide the fate of stem cells.^[^ ^53^, ^54^ ^]^ Although the TATO materials all had compressive modulus in the MPa range, TATO‐2 exhibited higher flexural properties, exhibiting a reduction of at least 12 times compressive modulus compared with TATO‐1, TATO‐3 and bulk PCL. It is widely acknowledged that in the context of BMSC, substrate stiffness surpassing 100 kPa tends to favor osteogenesis over neurogenesis and adipogenesis. The optimal stiffness range for adipogenesis is recognized to be ≈1 kPa, while for neurogenesis, it is ≈10 kPa. Therefore, while the TATO materials supported multilineage differentiations, the results suggest their potential suitability within the field of bone tissue engineering, possibly with, but not limited to, further bioconjugation with bone forming growth factors and osteoconductive bioceramics.^[^ ^55^, ^56^ ^]^

### 2.8 Fabrication Potential of the TATO Materials

**Figure 7:**
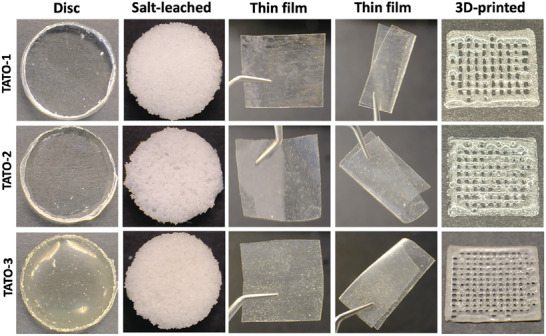
Different fabrication potential of the TATO materials illustrated by molded discs (degassed prior to curing to remove air bubbles), 3D porous solvent‐casting salt‐leaching scaffolds, thin films with flexible properties, and 3D‐printed structures.

The micro‐ and macro‐scale architectures of scaffolds play a crucial role in the success of tissue engineering and regeneration. These architectures guide the growth and differentiation of cells in contact, influencing the overall effectiveness of tissue regeneration.^[^ ^53^, ^57^, ^58^, ^59^, ^60^ ^]^ Therefore, biomaterials intended for scaffolding require high flexibility in formability. In the present study, the three TATO materials were fabricated in various scaffold forms for diverse tissue engineering applications. Prominently, the TATO materials could be shaped as molded discs by casting, 3D microporous spongy‐like scaffolds by solvent‐casting salt‐leaching technique, paper‐thin films, and porous grid structures by an extrusion‐based 3D printer (**Figure** **7**). The molded discs used in this study contained air bubbles, as they were fabricated immediately after mixing. These air bubbles would be efficiently removed by degassing the resin under vacuum prior to curing. Although the printability of the TATO materials was admittedly suboptimal with the extrusion‐based 3D printer, enhancements can be achieved by fine‐tuning the viscosity of the materials. This improvement can be accomplished, for instance, by incorporating bioceramics as fillers. Notably, the TATO materials were printed without a need of solvent at an ambient temperature, unlike conventional synthetic biopolymers such as PCL, PLA, and poly(trimethylene carbonate). This is particularly preferable in tissue engineering as the materials can be co‐printed with bioinks (i.e., cell‐laden hydrogels for bioprinting) without deteriorating the viability of the encapsulated cells and the adjunct biomaterials. Alternatively, we previously reported the successful 3D printing of materials with a similar chemical composition using a stereolithography (SLA) 3D printer. These findings highlight the potential of newly developed TATO‐based biomaterials, showcasing their versatile formability that can be applied across a range of potential applications in tissue engineering.^[^ ^61^ ^]^

## 3 Conclusion

In this study, we introduce TATO crosslinked thermosets as a novel class of materials suited for tissue engineering applications. The developed TATO technology possessed attractive properties that starkly distinguish them from conventional synthetic polymer scaffolding materials, such as high T_g_ polyesters or polycarbonates. The manageable viscosities of the resins at room temperature coupled with the rapid and complete photocuring via TEC and TYC click chemistry enabled the fabrication of discs, thin films, 3D conventional scaffolds, and 3D printed structures. This versatility highlights their promising role across a spectrum of biomedical applications. Particularly, the soft and flexible attributes of the TESTATO‐4PA‐based crosslinked thermoset material TATO‐2, along with the high tunability of the TATO materials through TEC or TYC chemistry, offer an unprecedented and promising alternative as potential scaffolding materials for tissue engineering. The developed TATO materials not only demonstrate support for cell attachment, growth, and multi‐lineage differentiation of BMSC on their surfaces but also exhibit comparable biocompatibility to medical‐grade PCL. This achievement paves a way to preclinical studies and, potentially, clinical translation.

## 4 Experimental Section

### Chemicals and Materials

Monomers 1,3,5‐triester‐4‐pentenoic acid‐1,3,5‐triazine‐2,4,6(1H,3H,5H)‐trione (TESTATO‐4PA) and 1,3,5‐triester‐4‐pentynoic acid‐1,3,5‐triazine‐2,4,6(1H,3H,5H)‐trione (TESTATO‐4PTYA) were synthesized as previously demonstrated.^[^ ^34^ ^]^ Monomer 1,3,5‐triallyl‐1,3,5‐triazine‐2,4,6(1H,3H,5H)‐trione (TATATO) and photoinitiator diphenyl(2,4,6‐trimethylbenzoyl)phosphine oxide (TPO) were purchased from Sigma‐Aldrich Sweden AB. Thiol monomer 1,3,5‐tris[2‐(3‐mercapto propionyloxy)ethyl] isocyanurate (TEMPIC) was obtained from Bruno Bock Chemische Fabrik GmbH & Co. KG. A teflon sheet (thickness: 1.5 mm) was purchased from RS components (Gothenburg, Sweden) and then processed into either disc‐shaped molds (diameter: 9 mm, thickness: 1.5 mm) or rectangular molds (length: 40 mm, width: 6.5 mm, thickness: 1.5 mm).

### Preparation of TATO Materials

The thermoset materials TATO‐1, TATO‐2 or TATO‐3 were fabricated by photo‐curing the thiol monomer TEMPIC with the alkene or alkyne monomers TATATO, TESTATO‐4PA or TESTATO‐4PTYA, respectively (**Table** **2**). The TEC or TYC materials were prepared as reported previously.^[^ ^34^ ^]^ The monomers were mixed, homogeneously, with TPO and then transferred to a disc‐shaped mold (9 mm in diameter, 1.5 mm thickness), unless mentioned otherwise, before exposure for photo‐initiated radical polymerization. For biological assessments in sterile conditions, the materials were sterilized by sequential washing twice in 75% ethanol and PBS before UV irradiation for 2 h.

**Table 2:** Thermoset materials evaluated in the study and the concentration of TPO for each material.

Materials	Alkene	Alkyne	Thiol	Equivalence alkene/alkyne to Thiol	TPO [wt%]
TATO‐1	TATATO	–	TEMPIC	1:1	0.25
TATO‐2	TESTATO‐4PA	–	TEMPIC	1:1	0.25
TATO‐3	–	TESTATO‐4PTYA	TEMPIC	0.5:1	0.88

### Rheological Testing

The rheological characteristics of the uncured resins for the three thermosets were examined using a DHR‐2 rheometer from TA Instruments. The measurements were conducted with a parallel geometry featuring a 20 mm diameter and a 300 µm gap. Flow‐sweep mode was employed to assess the viscosity‐ shear rate relationship. The shear rate varied from 10^−4^ to 10^2^ 1/s, with five data points per decade. The test was performed at 25 °C, and three different batches were evaluated for each resin. Raw data, collected in logarithmic scales (base 10), were analyzed using Trios software from TA Instruments.

### Surface Topographical Characterizations

For surface topographical analyses, SEM andAFM were performed. SEM images of the TATO materials were captured by FE‐SEM S‐4800 (Hitachi, Japan) after washing with Milli‐Q water. All samples were coated with a layer of Au‐Pt by a JFC 1300 Sputter Coater (JEOL). SEM images were captured with 15 kV acceleration voltage and 1 µA electron beam current, with 30× magnification. All captured images are provided in Figure S1, Supporting Information. The captured images were analyzed by ImageJ software (National Institutes of Health).^[^ ^37^ ^]^ The nano‐scale surface structures were characterized by AFM. Before image acquisition, specimens were washed in acetone under sonification to remove possible particles from the surfaces and then dried by air flow. The AFM images were captured using Bruker Multimode 8 with ScanAsyst Air mode (scanning rate: 512 points/line) in a square area of 5 × 5 µm^2^ on the surfaces of the discs. The height was rescaled at ±30 nm for direct comparison. For each material, a minimum of 5 specimens were analyzed.

### Contact Angle Measurement

Before the measurement, each specimen was rinsed with acetone on the surface and then dried by air flow. The wettability of the materials was evaluated using Contact Angle Meter (Biolin Scientific, theta lite model). During the measurement, a total volume of 4 µL Milli‐Q water was dropped on the center of the specimen and the contact angle was recorded by software OneAttension (version 4.1.6, r9978) with Young‐Laplace mode. The image recording setting was 10 s at 10% (Frame rate: 20 FPS). For each material, a minimum of 5 specimens were assessed.

### Raman Spectroscopy

A portable i‐Raman Plus spectrometer (model: BWS465‐785S, B&W TEK) was utilized to monitor possible chemical structure changes in the materials after incubation in PBS (pH = 7.4) at 37 °C. Before analysis, the specimens were washed with Milli‐Q water and dried in an oven at 50 °C overnight. The specimens were thereafter characterized with 48 scans (laser wavelength: 785 nm, laser power: 340 mW, integration time: 1000 ms). The data was collected on BWSpec software and then analyzed with Origin 2020. The carbonyl shift (1780 cm^−1^) was selected for the normalization of the spectra. The obtained Raman spectra for each material after 0, 1, 3, 7, and 14 days in PBS were compared to analyze potential changes in their chemical structures.

### Dynamic Mechanical Analysis

For dynamic mechanical analysis, specimens were prepared into rectangular beams with thickness of 1.5 mm, width of 6.5 mm and length of 20 mm. The glass transition temperatures (T_g_) and onset temperatures (T_onset_) of the TATO materials after immersion in PBS (pH = 7.4) for different durations were measured by a Dynamic Mechanical Analyzer (DMA Q800, T.A. Instruments, USA) in tensile mode. A temperature ramp method with a heating rate of 3 °C min^−1^ was used and the testing temperatures ranged from either −10, 0, and 10 °C to 80, 90, and 100 °C, depending on the materials’ properties. The strain and frequency were set to be 0.1% and 1 Hz, respectively. For each material, a minimum of 5 specimens were assessed.

### Compression Test

The mechanical properties of the TATO materials were assessed through compression testing. Samples at day 0 (under dry conditions) were immediately measured post‐material preparation. Samples were also measured after incubation in PBS at 37 °C for 1, 3, 7, and 14 days. Prior to testing, these samples were taken from the solution, rinsed with Milli‐Q water, before excess water was removed with a tissue. An Instron 5566 universal testing machine (Instron Korea LLC) was used with a 10 kN load cell. For all the experiments, the compression rate was set at 1 mm min^−1^, and tests concluded at either a 100% compressive strain or a compressive load of 10 kN. All tests were conducted at 20 °C and a relative humidity of 50%. Bluehill software was employed for data analysis and collection. For each material, a minimum of 5 specimens were assessed.

### In Vitro Biodegradation Test of TATO Materials

Disc shaped specimens of the TATO materials were incubated in the presence of porcine carboxyl esterase (1 mg mL^−1^; E3019, Sigma‐Aldrich, USA), porcine triacylglycerol lipase (1 mg mL^−1^; E3126, Sigma‐Aldrich, USA) or PBS supplemented with 1% penicillin and streptomycin for 14 days in 5% CO_2_ humidified atmosphere at 37 °C. During incubation, a sterile environment was maintained. To determine their dry weight, liquid was removed from the specimens through centrifugation at 300 rcf for 5 min followed by vacuum drying at 37 °C for 45 min.

### Cell Culture and Seeding

Fibroblastic cell line L929 (American Type Culture Collection CCL‐1) were cultured in Dulbecco's Modified Eagles Medium (DMEM: 21885‐025; Gibco, USA) supplemented with 10% fetal bovine serum (FBS: 10270‐106; Gibco, USA) and 1% penicillin and streptomycin (PS: SV30010; HyClone, USA) in 5% CO_2_ humidified atmosphere. Furthermore, the effects of the various materials on the differentiation of humanBMSC were determined. Human BMSC were isolated and characterized as previously described under ethical clearance (2020/7199/REK sør‐øst C).^[^ ^62^ ^]^ The BMSC were maintained in α‐minimum essential medium (α‐MEM: 22 571; Gibco, USA) supplemented with 10% FBS and 1% penicillin and streptomycin in a 5% CO_2_ humidified atmosphere. Cells from passage 4–5 derived from two donors were used for the experiments.

### Indirect Cytotoxicity Tests

Cytocompatibility of the TATO was evaluated by indirect cytotoxicity tests, namely an agar diffusion test and a MTT (3‐(4,5‐Dimethylthiazol‐2‐yl)−2,5‐Diphenyltetrazolium Bromide) assay, using L929 fibroblasts in accordance with ISO 10993–5:2009(E).^[^ ^63^ ^]^ Detailed methodology is available in the supplementary information.

### Direct Cytocompatibility Tests

Large discs of TATO‐1, TATO‐2 and TATO‐3 were prepared with a diameter of 35 mm. L929 cells (1 × 10^5^) were resuspended in 600 µL of culture medium and seeded on top of each disc (*n* = 2 for each material). Cells were allowed to attach for 1 h in 5% CO_2_ humidified environment before adding 2.4 mL additional medium. After 3 days of incubation, the cells were resuspended, and the viability of cells and apoptosis were evaluated by the FITC‐Annexin V Apoptosis Detection Kit II (556 570; BD Biosciences, USA) following the manufacturer's protocol, using a flow cytometer (BD Accuri C6 Flow Cytometer, BD Biosciences, USA). The data was analyzed using flow cytometry data analysis software (FlowJo V10, Flowjo, USA). Additionally, BMSC were seeded directly onto the various TATO discs and cultured in the osteogenic medium for 7 days. MTT assay was performed as aforementioned with *n* = 5 for each material, and the experiment was independently repeated twice.

### Cell Morphology and Attachment on TATO Materials

TATO discs were cultured with L929 (*n* = 2) cells and BMSC (*n* = 2). Then, cells were fixed in 3% glutaraldehyde (1 042 390 250; Merck, USA) in 0.2 m Na‐cacodylate buffer (C0250; Merck, USA) with sucrose (S0389; Merck, USA), dehydrated in graded ethanol solutions, sputter coated with a layer of Pd/Au using a high‐resolution fine coater (JFC‐2300HR, Jeol, Japan) and imaged using SEM (JSM‐7400F, Jeol, Japan). Attachment and morphology were also evaluated through cytoskeletal staining by Phalloidin (1:250, A12379; Invitrogen, USA) and 4',6‐diamidino‐2‐phenylindole (DAPI, 1:2500, D9542; Sigma‐Aldrich, USA). Image acquisition was performed on a confocal laser microscope (TCS SP8, Leica, Germany).

### Multi‐Lineage Differentiation of BMSC

To evaluate the effects of TATO materials on the multi‐linage of BMSC, the cells were cultured on the TATO discs as described above and then induced into osteogenic, adipogenic, and neurogenic lineages. For osteogenic differentiation, BMSC were cultured on the various TATO discs at a density of 8000 cells/disc for 14 days in the osteogenic medium, consisting of α‐MEM with 10% FBS and 1% PS supplemented with 10 nm dexamethasone (D4902; Sigma, USA), 10 mm beta‐glycerophosphate (G9422; Sigma, USA), and 173 µm L‐ascorbic acid (A8960; Sigma, USA) while for adipogenic differentiation, the cells were maintained in medium supplemented with 10 nm Dexamethasone, 10 µg mL^−1^ Insulin (I9278‐5ML; Sigma, USA), 0.2 mm Indomethacin (17 378–5G; Sigma, USA) and 0.5 mm 3‐Isobutyl‐1‐methylxanthine (IBMX) (I7018‐100MG; Sigma, USA). Mineral deposition for osteogenic differentiation and fat droplets for adipogenic differentiation were evaluated by alizarin red S staining and Oil Red O staining, respectively, as previously described.^[^ ^62^ ^]^ For neurogenic differentiation, the cells were induced by Mesenchymal Stem Cells Neurogenic Differentiation Medium (C‐28015; PromoCell, Sigma‐Aldrich, USA) in accordance with the manufacturer's protocol.

### Cytochemical and Immunofluorescent Staining

The samples were fixed in 4% PFA for 15 min followed by permeabilization in 0.1% Triton X‐100 in PBS for a further 10 min at room temperature, except for the samples for collagen type1 (Col1) staining where the samples were fixed in ice‐cold methanol for 10 min. After 60 min of blocking in 10% Normal Goat Serum (NGS) in 0.1% Triton X‐100 in PBS at room temperature, the samples were stained with the following primary antibodies at 4 °C overnight. The primary antibodies used were: anti‐MAP2 Polyclonal antibody (1:250, PA5‐17646; ThermoFischer, USA), anti‐Beta‐III tubulin (1:250, 53‐4510‐82; ThermoFischer, USA) and anti‐Col1 (1:250, EPR7785; Abcam, UK). For non‐conjugated antibodies, secondary incubation with Goat Anti‐Rabbit IgG conjugated with AlexaFluor 635 antibody (A31576, ThermoFischer, USA) was performed for 1 h at room temperature. Nuclei and F‐actin were counterstained with DAPI (1:2500; D9542; Sigma‐Aldrich, USA) and Phalloidin (1:250, A12379; Invitrogen/1:250, 94 072; Sigma‐Aldrich, USA). Fluorescent images were acquired using the laser confocal microscope equipped with a water immersion 20× objective. The images were processed using Fiji/ImageJ.

### Fabrication of Scaffolds for Tissue Engineering Applications

In addition to the discs described above, the formability of the TATO‐based materials was evaluated by fabricating membranes and 3D porous scaffolds using the solvent‐casting salt‐leaching technique and 3D‐printing. The thin membranes were fabricated using a 200 µm film applicator before photo‐curing. Solvent‐casting salt‐leaching scaffolds were fabricated as previously described.^[^ ^64^ ^]^ Briefly, TATO monomers were mixed with sodium chloride particles, with a size ranging from 90 to 600 µm, and acetone and casted in a glass dish. The acetone was allowed to be evaporated before photo‐curing, and disc‐shaped scaffolds were punched into a diameter of 10 mm before the salt particles were leached in distilled H_2_O. 3D printed scaffolds were fabricated using an extrusion‐based 3D‐printer (3D Bioplotter RP, EnvisionTEC, Germany). Magics software (EnvisionTEC, Germany) was used to design a 3D CAD model of a box measuring 15 mm in diameter, sliced into layers at a slice thickness of 80% the inner diameter of the extrusion needle (i.e., 500 µm). Two layers were printed. The distance between the strands ranged from 1.4–1.6 mm. The materials were printed at 20–22 °C and light cured with a handheld dental curing lamp (Bluephase PowerCure, Ivoclar Vivadent, Liechtenstein, dominant wavelengths of 400 and 470 nm) at the highest intensity (2000 mWcm^−2^).

### Statistical Analysis

All data are presented as mean with standard deviation (SD) in parentheses. Origin 2020 (academic, USA) and Prism 9 (Dotmatics, USA) were used to perform statistical analysis. One‐way ANOVA followed by Tukey's multiple comparisons test was used for the degradation studies (*n* = 5 per group) and MTT indirect test on extract (*n* = 4). Two‐way ANOVA followed by Tukey's test was used for MTT direct test with BMSC (*n* = 5). A *p* < 0.05 was considered to be statistically significant in all the biocompatible testing.

## Conflict of Interest

The authors declare no conflict of interest.

## Supporting information

Supporting Information

## Data Availability

The data that support the findings of this study are available from the corresponding author upon reasonable request.
