# Supplementary material for: Photo‐Clickable Triazine‐Trione Thermosets as Promising 3D Scaffolds for Tissue Engineering Applications
Source: Adv Healthc Mater. 2024 Jul 17;13(27):2401202. doi: 10.1002/adhm.202401202 (PMC12344621; doi:10.1002/adhm.202401202)
Supplement: Supplementary file 1 — Supporting Information [file ADHM-13-0-s001.docx]

Supporting Information

**Photo-Clickable Triazine-Trione Thermosets as Promising 3D Scaffolds for Tissue Engineering Applications**

*Åshild Johansen^§^, Jinjian Lin^§^, Shuntaro Yamada, Samih Mohamed-Ahmed, Mohammed A. Yassin, Cecilie Gjerde, Daniel J. Hutchinson, Kamal Mustafa^*^ and Michael Malkoch^*^*

*^§^ Equal contribution*

** Correspondence authors*

**Supplementary Materials and Methods**

Agar diffusion test.

10 ml of 1% agar solution consisting of melted agar (Agar: 9002180; Sigma-Aldrich, USA) in milli-Q water and 2X DMEM (SLM-202-B; Sigma-Aldrich, USA) supplemented with 20% FBS and 2% PS was poured into a 100 mm petri dish where L929 cells were seeded in a density of 18000 cell/cm^2^ and grown until 80% confluency. After gelation, 10 ml of 2.5% neutral red (NR: N4638-5G; Sigma Aldrich, USA) in PBS was added and incubated in a 5% CO_2_ humidified atmosphere for 30 minutes. The NR solution was removed, and TATO discs of diameters 9 mm (n=6) were placed on the solidified agar layer. Polycaprolactone (PCL: Resomer C 212, Evonik, Germany) and latex served as negative and positive controls, respectively. After 24 hours of incubation in a 5% CO_2_ humidified atmosphere at 37 ℃, cytotoxic effects of the TATO samples were evaluated macroscopically and microscopically (Nikon Eclipse Ti, Japan). The grade for cytotoxicity was evaluated in a scale shown in **Table S1**.

**Table S1**. The grade scale of the agar diffusion test.

| **Grade** | **Reactivity** | **Description of reactivity zone** |
| --- | --- | --- |
| 0 | None | No detectable zone around or under specimen |
| 1 | Slight | Some malformed or degenerated cells under specimen |
| 2 | Mild | Zone limited to area under specimen |
| 3 | Moderate | Zone extending specimen size up to 1.0 cm |
| 4 | Severe | Zone extending farther than 1.0 cm beyond specimen |

***MTT assay.***

Extracts from the materials were prepared by conditioning the growth medium in the TATO materials and PCL discs with a ratio of 3 cm^2^/ml for 24 hours in a 5% CO2 humidified environment at 37 °C (40). Control samples containing only medium were treated similarly.

L929 cells were seeded in a density of 1 × 10^4^ cells per well in a 96 well-plate and maintained in culture for 24 hours. After incubation, the culture medium was aspirated and 100 µl of the extract solution or complete growth medium as a negative control were added for another 24 h. A 1 mg/ml MTT (Thiazolyl Blue Tetrazolium Bromide; 298-93-1; Sigma-Aldrich, USA) was then added to the plates and incubated further for 3 hours. After removing MTT solution, purple formazan products were dissolved in a solubilization solution consisting of DMSO with 6.25% 0.1 M NaOH on a shaker for 20 minutes at room temperature. The absorbance was measured at 570 nm using a VarioskanTM LUX multimode microplate reader (VLBL00D0: Thermo Scientific, Finland). The experiment was conducted twice (n=10 for each time).

**Supplementary results**


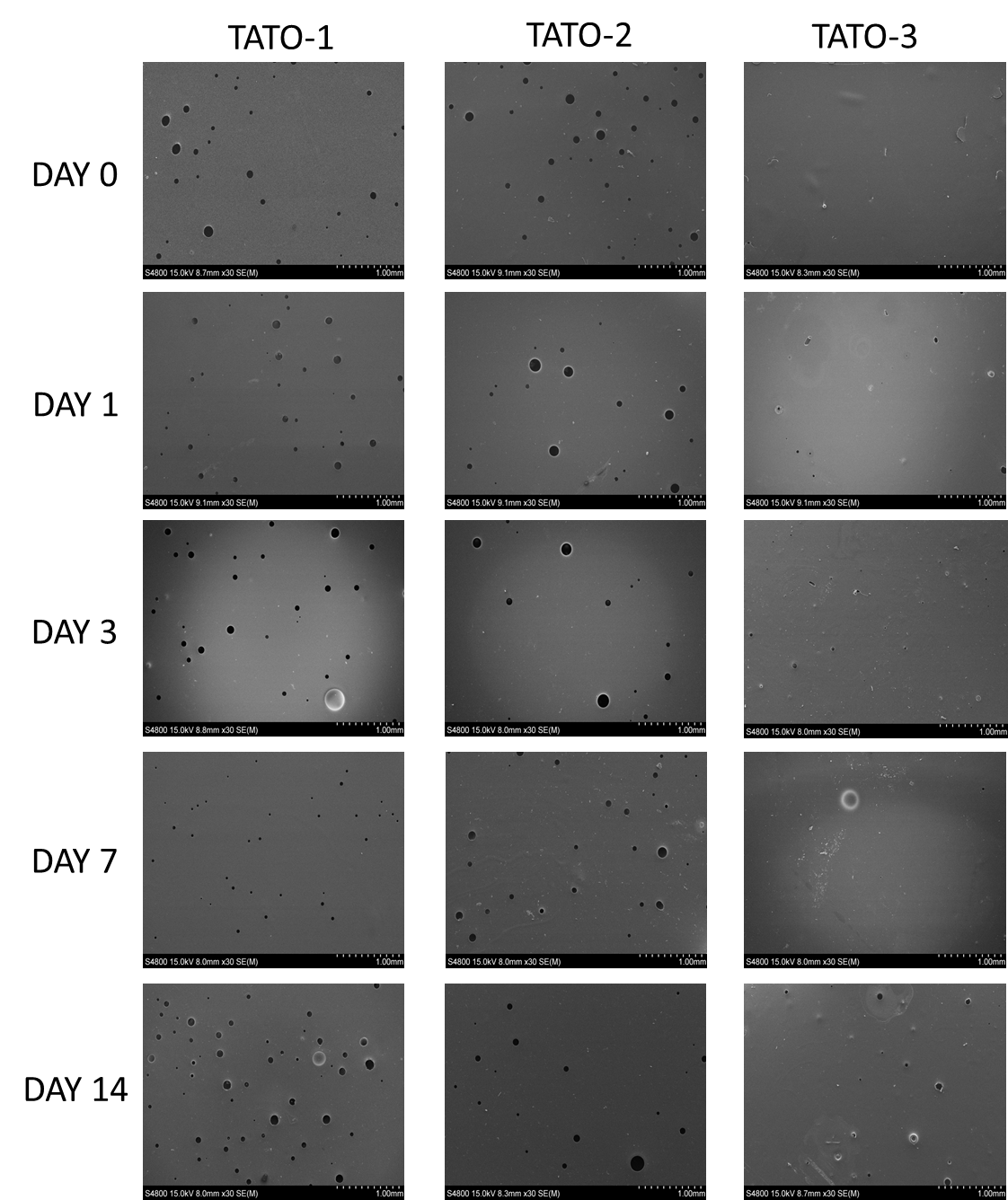


Figure S1. SEM images of the surfaces of of each TATO material before and after 1 day, 3 days, 7 days and 14 days incubation in PBS (pH=7.4).


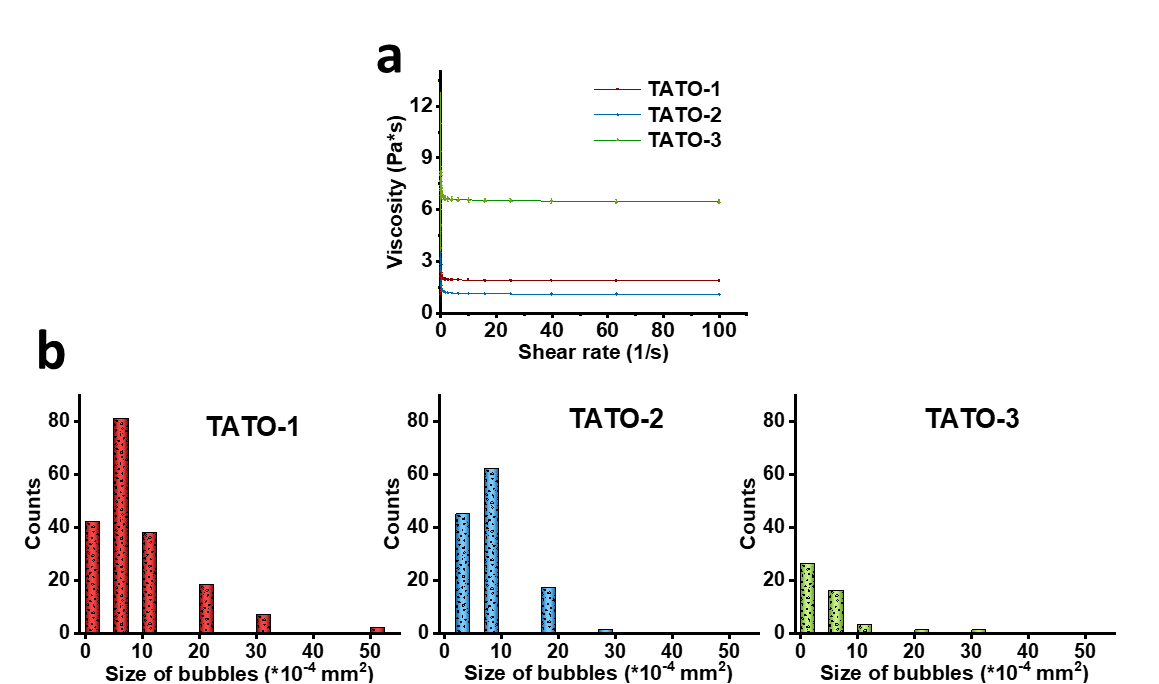


Figure S2. a). Rheological results of the TATO resins at room temperature; b) Histogram of air bubble size distribution on the surface of the discs from SEM images of each material after different time points in PBS (pH=7.4). n=5 for each material.

**
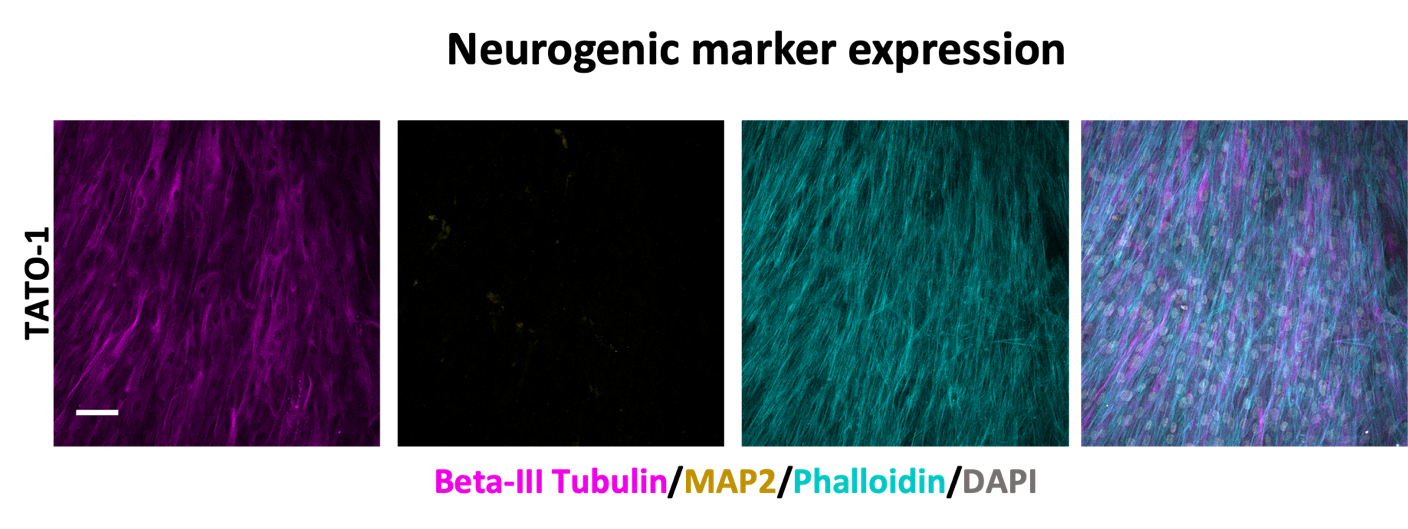
**

Figure S3. Neurogenic marker expression of bone marrow mesenchymal stem cells (BMSC) in the growth medium. Immunostaining shows the positive expression of Beta-III Tubulin, but not MAP2. Phalloidin and DAPI were used for counterstaining of the cytoskeleton and nuclei. BMSC on TATO-1 was displayed as control for the neurogenic marker expression. Scale bar = 100 µm.
